# Supplementary figures and images for: Moderate and intensive mechanical loading differentially modulate the phenotype of tendon stem/progenitor cells in vivo
Source: PLoS One. 2020 Dec 29;15(12):e0242640. doi: 10.1371/journal.pone.0242640 (PMC7771689; doi:10.1371/journal.pone.0242640)

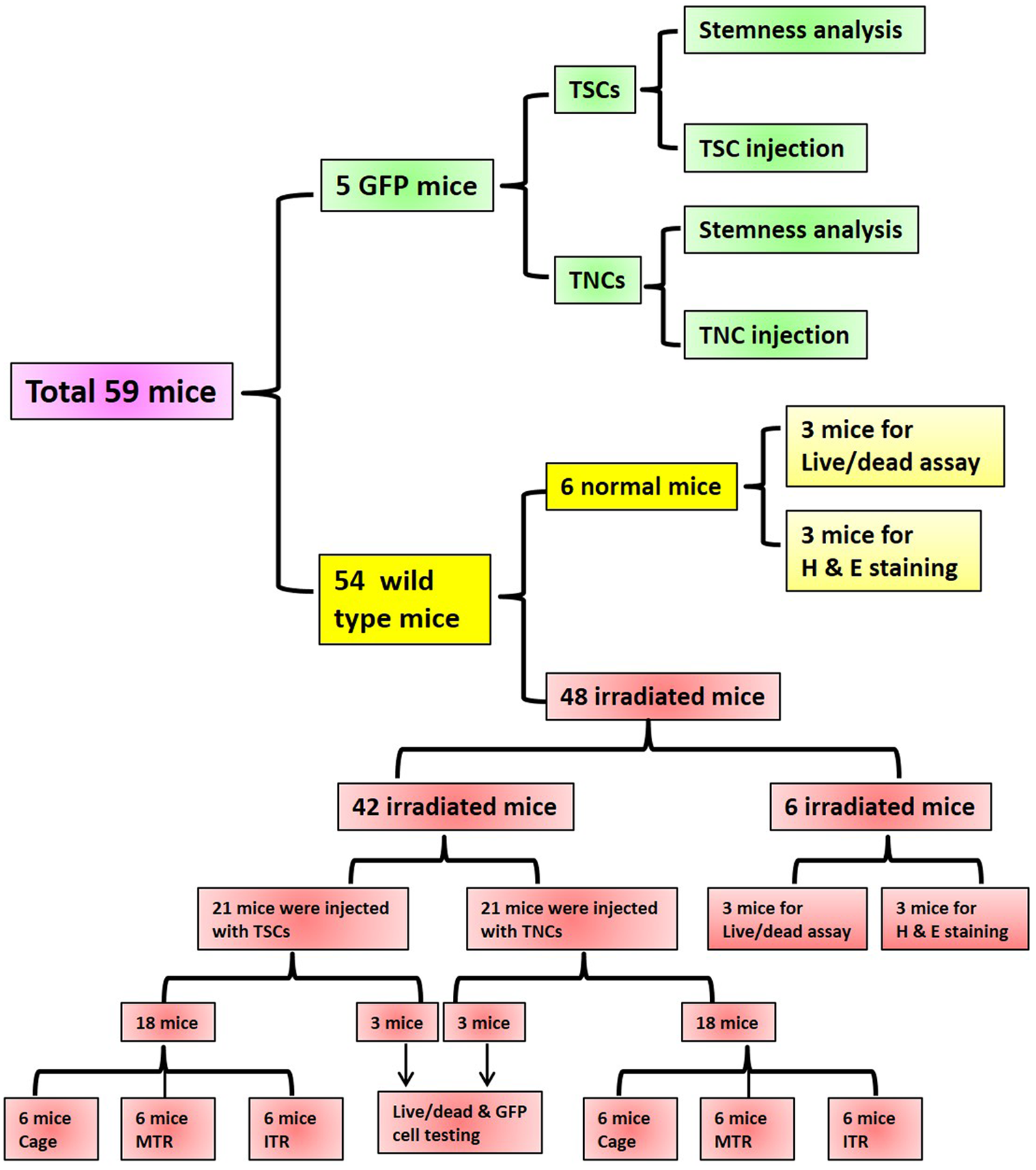

Supplement: S1 Fig — (TIF) [file pone.0242640.s001.tif]
