# Supplementary material for: Moderate and intensive mechanical loading differentially modulate the phenotype of tendon stem/progenitor cells in vivo
Source: PLoS One. 2020 Dec 29;15(12):e0242640. doi: 10.1371/journal.pone.0242640 (PMC7771689; doi:10.1371/journal.pone.0242640)
Supplement: Attachment — Submitted filename: Response to Reviewers_12_01_20_SV.docx [file pone.0242640.s002.docx]

Response to Reviewers’ Comments

**Reviewer #1**

General Comment: In this papers Authors evaluated the effect of TSCs and TNCs injection in irradiated mice after MTR and ITR. The study is interesting but some concerns need to be addresses

**Response:** Thank you for your comments.

**Specific Comments**

1. Authors should submit a higher resolution figure 3. Additionally, nuclear staining with DAPI or HOECHST is required to clarify the presence of cells.
   The same observation applies to figure 7.

   **Response:** We have provided new figures 3 and 7, with a higher resolution. DAPI staining has been added to show the presence of cells, as requested.
2. In the discussion Authors state that in their study they demonstrate TSCs are responsible for the development of tendinopathies during ITR through aberrant differentiation. However, Authors exclusively compare the differentiation capacities of TSCs and TNCs during MTR and ITR. So, the conclusions need to be revised. To confirm what they stated, it would also be necessary to evaluate hypercellularity, hypervascularity and expression of inflammatory agents.

**Response:** The purpose of this study was to determine the differential mechanobiological responses between TSCs and TNCs. We found that in response to ITR but not MTR, TSCs but not TNCs were able to differentiate into non-tenocytes. Based on this finding, we speculated that TSCs may be responsible for the development of tendinopathy. But keep in mind that since the mechanical overloading (ITR) was relatively short (3 weeks), it is likely that the state of tendinopathy was just in an early developing stage. To achieve a full-blown development of tendinopathy, which exhibits the said characteristics – hypercellularity, hypervascularity, and expression/production of inflammatory agents, a much longer time period is required. Indeed, maturation of tendinopathy takes years of mechanical overloading placed on the tendon. We have revised the related text to make this point clear.

Reviewer: 2

General Comment: The manuscript is very interesting; figures are good. The Authors demonstrated that GFP-TSC injected patellar tendons within ITR mice exhibited rounding of cells, elevated chondrogenic differentiation, and elevated gene expression and immunostaining of non-tenocyte markers. Although this, the authors should address some concerns.

Response: Thank you for your summary of this study.

Specific Comments

1. The results are overinterpreted when the authors state that resident TSCs, but not TNCs, contribute to the development of ITR-induced tendinopathy through aberrant differentiation. In my opinion in order to better characterize the type of tendinopathy, the authors must evaluate other parameters such as inflammation, immunomodulating factors, proliferation and tendon disintegration. In fact, in the phase of reactive or acute tendinopathy there is an accumulation in loco of cytokines and immunomodulating factors that cause an initial inflammation with cell proliferation. The transition from the reactive tendinopathy phase to that of degenerative tendonitis corresponds to the failed healing phase (failure of the self-healing process) which causes tendon disintegration. The tendon, at the end of this process, will have degenerated portions that become unable to develop tensile strength. Therefore, the Authors must change the conclusions or add experiments that confirm their hypotheses.
   In addition, the manuscript must be revised by a native English speaker.

**Response:** As explained in our response to reviewer #1 above, our study focused on how TSCs respond differently from TNCs. We showed that TSCs but not TNCs were able to differentiate into non-tenocytes under a short term, or 3 weeks of mechanical overloading condition. We suggest that our results are from early developing stage of tendinopathy, whereas what the reviewer described are characteristics of later stage of tendinopathy, mostly in humans. We have clarified the related text in the revision.

In terms of the need for a native English speaker for revision of this manuscript, the authors are not clear about the specific aspects of English usage problems which are referred in the reviewer’s comment. Indeed, there are a few native English speakers (KW, AM, MH) who participated in drafting this manuscript. Nevertheless, we have closely revised the entire manuscript.
